# An application of the Continuous Opinions and Discrete Actions (CODA) model to adolescent smoking initiation

**DOI:** 10.1371/journal.pone.0186163

**Published:** 2017-10-11

**Authors:** Ruoyan Sun, David Mendez

**Affiliations:** Department of Health Management and Policy, University of Michigan, Ann Arbor, MI, United States of America; Universite Toulouse 1 Capitole, FRANCE

## Abstract

We investigated the impact of peers’ opinions on the smoking initiation process among adolescents. We applied the Continuous Opinions and Discrete Actions (CODA) model to study how social interactions change adolescents’ opinions and behaviors about smoking. Through agent-based modeling (ABM), we simulated a population of 2500 adolescents and compared smoking prevalence to data from 9 cohorts of adolescents in the National Survey on Drug Use and Health (NSDUH) from year 2001 till 2014. Our model adjusts well for NSDUH data according to pseudo *R*^2^ values, which are at least 96%. Optimal parameter values indicate that adolescents exhibit imitator characteristics with regard to smoking opinions. The imitator characteristics suggests that teenagers tend to update their opinions consistently according to what others do, and these opinions later translate into smoking behaviors. As a result, peer influence from social networks plays a big role in the smoking initiation process and should be an important driver in policy formulation.

## Introduction

Tobacco use has been a focus of public health interventions for decades but still remains as the single largest preventable cause of disease and premature death in the US and around the world [1, 2]. Studies have shown that tobacco consumption can lead to adverse health consequences such as heart disease, many types of cancer, pulmonary disease, adverse reproductive outcomes and exacerbation of multiple chronic health conditions [3]. Tobacco consumption is the primary causal factor for at least 30% of all cancer deaths, for nearly 80% of deaths from chronic obstructive pulmonary disease, and for early cardiovascular diseases and deaths [1]. In addition, in the US, cigarette smoking alone has been estimated to cause 443,000 deaths per year, including approximately 49,400 deaths attributable to secondhand smoke exposure [4]. Smoking also creates a financial burden for society as a whole. It is estimated that cigarette smoking costs the US $96 billion in direct medical expense and $97 billion in lost productivity per year [4]. And it is estimated that by 2010, 8.7% of annual healthcare spending in the US was attributable to smoking, that is a total of $170 billion annually [5].

Researchers have discovered that smoking behavior is determined by many factors such as age, gender and socioeconomic status [6]. There is evidence that influence from social interaction plays a big role on teenager smoking behavior and, in particular, on the smoking initiation process [7–9]. Freedman et al. conducted a systematic review on smoking initiation among young adults in the US and Canada from 1998 to 2010. They found consistent evidence that teenagers have a higher risk to start smoking if they are exposed to it [10].

Despite the positive association established by previous literature between peer influence and smoking initiation, very few studies have attempted to investigate the underlying mechanism. One approach to study the spread of peer influence is through opinion dynamics. Opinion dynamics is a field created by statistical physicists to study how simple contact rules of individuals can explain the diffusion of opinions among the population. Models in opinion dynamics use quantitative methods such as computer simulation to model diffusion or propagation of opinions across individuals in the population. Opinion dynamics has many applications in social science with regard to formation of opinions [11]. One recent study from Moore et al. presented a social-network-based opinion dynamics model to map addictive health behaviors [12].

Opinion Dynamics models can be implemented and tested through computer simulations, which has been proved to be useful in informing tobacco policies [13]. A relatively new tool in the social science research arsenal, agent-based modeling (ABM) has enabled scholars to investigate behaviors and social interactions at individual or organizational levels [14, 15]. ABM can incorporate various individual and environmental characteristics into the model to account for population heterogeneity. For example, Karimi et al. used ABM to study the effect of individual behaviors on flu transmission [16]. In addition, ABM takes social interactions and the corresponding impact on individuals into consideration. In their report of assessing the use of ABM for tobacco regulation, the Institute of Medicine (IOM) recommends the use of ABM and believes it to be a useful tool to further our understanding of smoking behaviors [17]. Many researchers have adopted ABM to investigate social and health issues as well as to evaluate policies. Hammond and Ornstein used ABM to build a model of social influence on body weight to predict future obesity trends [18]. And Hennessey et al. evaluated and refined an obesity intervention through ABM [19].

In this paper, we aim to investigate the impact of people’s opinions on the smoking initiation process among adolescents. Previous literature has established that smoking initiation is determined by different factors including social interactions and individual characteristics. For instance, the Oklahoma Communities of Excellence in Tobacco Control (CX) program used community-based practice to change social norms of smoking and local counties experienced positive changes with regard to smoking behaviors [20]. However, to the best of our knowledge, there are few papers that offer a quantitative framework to study the influence of peers on smoking initiation process.

We plan to use the Continuous Opinions and Discrete Actions (CODA) model developed by AC Martins as the underlying mechanism of opinion diffusion [21]. The CODA model consists of three parts: 1) CODA models opinions as unobservable and continuous variables bounded between 0 and 1; 2) individuals have binary actions that are observable; 3) individuals update opinions by incorporating peer behavior using a Bayesian update rule. Martins applied the CODA model to explain the emergence of extreme opinions in the population [22].

The organization of this paper is as follows: In section 2, we introduce the theoretical CODA model under the context of smoking initiation; Section 3 includes details of the model and parameter optimization based on empirical data; main results of the simulation are shown in Section 4; we present some qualitative analysis of likelihoods with respect to opinion change in Section 5 and discuss implications of this study in Section 6.

## Methods

Each individual in our model faces a binary decision of smoking (*S*) or non-smoking (*N*). We assume that each individual has a personal value function where the individual assigns a real number to the two options: smoking and non-smoking. We can write the value function for individual *i* at time *t* mathematically as: *f*_*i*,*t*_: {*S*, *N*} → *R*. When making the decision regarding smoking, *i* compares the values assigned to both options. Consequently if *i* values smoking more than non-smoking, *i* has the opinion that smoking is better than non-smoking. If *i* values non-smoking more, then he or she has the opinion that non-smoking is the better alternative. We can map these two opinions for each individual at time *t* onto the [0, 1] interval. We treat them as probabilities because opinions do not translate into behaviors 100%. Let *p*_*i*,*t*_ = *Pr*(*f*_*i*,*t*_(*S*) ≥ *f*_*i*,*t*_(*N*)) and it follows that 1 − *p*_*i*,*t*_ = *Pr*(*f*_*i*,*t*_(*S*) ≤ *f*_*i*,*t*_(*N*)). Here *p*_*i*,*t*_ and 1 − *p*_*i*,*t*_ reflect the continuous opinions that *i* holds at time *t*, which is not directly observable.

As in the initial description of the CODA model [21], individuals are able to update their opinions at every time step by observing their peers’ behavior. For example, if *i* observes that peers smoke at time *t*, then opinion at time *t* + 1, *p*_*i*,*t*+1_, will change depending on initial opinion and likelihoods. We define a discrete choice space *δ*_*i*,*t*_ for *i* where

$$\begin{array}{c} \delta_{i,t}(p)=\{\begin{array}{cc} +1, & \text{if}\;i\;\text{chooses}\;S\;\text{at}\;t\\ -1, & \text{if}\;i\;\text{chooses}\;N\;\text{at}\;t \end{array} \end{array}$$ (1)

We also need likelihoods to implement the Bayesian update. Likelihoods are probability assessments made by individuals before any interaction takes place. The theoretical model implemented here is an extension of the original CODA paper to incorporate non-symmetrical likelihoods [21]. We denote *α*_*i*,*j*_ to be the likelihood that *i* assign to *j* that *j* is a smoker conditional on *i* valuing smoking more than non-smoking. The likelihood is a necessary part of the classic Bayesian update model. We write it in the following mathematical form:

$$\begin{array}{c} \alpha_{i,j}=Pr(\delta_{i,j}=+1|f_{i,t}(S)\geq f_{i,t}(N)). \end{array}$$ (2)

1 − *α*_*i*,*j*_ is the likelihood *i* assigns to *j* that *j* is not a smoker conditional on *i* having a higher value for smoking. Similarly, we have *β*_*i*,*j*_ = *Pr*(*δ*_*i*,*j*_ = −1|*f*_*i*,*t*_(*S*) ≤ *f*_*i*,*t*_(*N*)), meaning the likelihood *i* assigns that neighbor *j* is a non-smoker, conditional on non-smoking is preferred by *i*. And 1 − *β*_*i*,*j*_ is the likelihood *i* assigns that *j* is a smoker, conditional on *i* valuing non-smoking more. We assume that all individuals in this model share the same likelihoods across time and drop the index for *α* and *β*.

Based on Bayesian update, we can infer the updated probabilities of *i* holding the opinion that smoking is better than non-smoking after interacting with a smoker from initial priors and likelihoods *α* and *β*.

$$\begin{array}{c} \begin{array}{cc} Pr(f_{i,t}(S)\geq f_{i,t}(N)|\delta_{j,t}=+1) & =\frac{Pr(f_{i,t}(S)\geq f_{i,t}(N),\delta_{j,t}=+1)}{Pr(\delta_{i,j}=+1)}\\ & =\frac{\frac{Pr(f_{i,t}(S)\geq f_{i,t}(N),\delta_{j,t}=+1)}{Pr(f_{i,t}(S)>f_{i,t}\left(N\right))}Pr(f_{i,t}(S)\geq f_{i,t}\left(N\right))}{Pr(\delta_{i,t}=+1)}\\ & =\frac{Pr(\delta_{j,t}=+1|f_{i,t}(S)\geq f_{i,t}(N))Pr(f_{i,t}(S)\geq f_{i,t}(N))}{Pr(\delta_{j,t}=+1)}\\ & =\frac{\alpha Pr(f_{i,t}(S)\geq f_{i,t}(N))}{Pr(\delta_{j,t}=+1)}\\ & =\frac{\alpha p_{i,t}}{Pr(\delta_{j,t}=+1)} \end{array} \end{array}$$ (3)

Similarly, we can write the other three probabilities as the following:

$$\begin{array}{c} \begin{array}{cc} & Pr(f_{i,t}(S)\geq f_{i,t}(N)|\delta_{j,t}=-1)=\frac{(1-\alpha )p_{i,t}}{Pr(\delta_{j,t}=-1)}\\ & Pr(f_{i,t}(S)\leq f_{i,t}(N)|\delta_{j,t}=+1)=\frac{\left(1-\beta )(1-p_{i,t}\right)}{Pr(\delta_{j,t}=+1)}\\ & Pr(f_{i,t}(S)\leq f_{i,t}(N)|\delta_{j,t}=-1)=\frac{\beta (1-p_{i,t})}{Pr(\delta_{j,t}=-1)} \end{array} \end{array}$$ (4)

We almost know the values of these posterior opinions given priors and likelihoods except we do not have the values for constants *Pr*(*δ*_*j*,*t*_ = +1) and *Pr*(*δ*_*j*,*t*_ = −1). We can get rid of these unknown constants by taking odds of the probabilities.

$$\begin{array}{c} \begin{array}{cc} & O(f_{i,t}(S)\geq f_{i,t}(N)|\delta_{j,t}=+1)=\frac{Pr(f_{i,t}(S)\geq f_{i,t}(N)|\delta_{j,t}=+1)}{Pr(f_{i,t}(S)\leq f_{i,t}(N)|\delta_{j,t}=+1)}=\frac{\alpha p_{i,t}}{\left(1-\beta )(1-p_{i,t}\right)}\\ & O(f_{i,t}(S)\geq f_{i,t}(N)|\delta_{j,t}=-1)=\frac{Pr(f_{i,t}(S)\geq f_{i,t}(N)|\delta_{j,t}=-1)}{Pr(f_{i,t}(S)\leq f_{i,t}(N)|\delta_{j,t}=-1)}=\frac{(1-\alpha )p_{i,t}}{\beta (1-p_{i,t})} \end{array} \end{array}$$ (5)

Here the term, *O*(*f*_*i*,*t*_(*S*) ≥ *f*_*i*,*t*_(*N*)|*δ*_*j*,*t*_ = +1), is the posterior odds of smoking for *i* after observing neighbor *j* is a smoker. And *O*(*f*_*i*,*t*_(*S*) ≥ *f*_*i*,*t*_(*N*)|*δ*_*j*,*t*_ = −1) stands for the posterior odds of smoking for *i* after observing *j* is a non-smoker. We then derive the posterior opinion of smoking for *i* from these odds ratio,

$$\begin{array}{c} p_{i,t+1}=\{\begin{array}{cc} \frac{O(f_{i,t}(S)\geq f_{i,t}(N)|\delta_{j,t}=+1)}{1+O(f_{i,t}(S)\geq f_{i,t}(N)|\delta_{j,t}=+1)}, & \text{if}\;j\;\text{is}\;\text{a}\;\text{smoker}\\ \frac{O(f_{i,t}(S)\geq f_{i,t}(N)|\delta_{j,t}=-1)}{1+O(f_{i,t}(S)\geq f_{i,t}(N)|\delta_{j,t}=-1)}, & \text{if}\;j\;\text{is}\;\text{a}\;\text{non-smoker} \end{array} \end{array}$$ (6)

## Simulations

We set the initial space to be a 50 × 50 grid representing 2500 individuals and measure the prevalence of smokers in the population over time. In our simulation, we consider the time step to be one month. For all individuals in the grid, we assume they share the same initial opinion. That means $p_{i,t_{0}}=p(0)$ for all *i*. We also implement a time invariant cessation rate and set it to be 4.2% per month for smokers who update their opinions as found in [23]. That means, smokers who update their opinions have a probability of 4.2% to stop smoking at every time step *t*. If they quit smoking, they reenter the pool of potential smokers in a month.

Then we adopt an interaction rule called random interaction. Under random interaction, an individual *i* is randomly picked from the population at every time step to interact with another randomly picked individual *j* from the population, here *i* ≠ *j*. Through observing *j*’s behavior, i then updates his or her opinion with regard to smoking. We repeat this process for 10% of the population every month.

With empirical data from the National Survey on Drug Use and Health (NSDUH) on cigarette use (prevalence) among adolescents between 12 and 17 from year 2001 to 2014, we are able to reveal the mechanism that teenagers interact with each other using the proposed model. We have 9 cohorts of teenagers from age 12 to 17 starting in 2001 till 2009. We assume that we model a monthly behavior change and obtain a total of 72 data points for each round of simulation. Due to the randomness involved in the interaction process, we run 200 rounds of simulation for each data point. We then take the average of all 200 rounds of simulation. From average values, we compare 6 data points (beginning of each year) with the empirical data. In order to find optimal parameter values that fit the real data, we search for the combination of parameters that minimize the difference squared between these two series. We conducted the search by examining all possible combinations of alpha and beta accurate to two decimal places.

## Results

We obtained results in Table 1 by computer simulations of ABM. The first row shows the corresponding cohort names, which are in chronological order. The second through fourth rows provide values of parameters under random interaction. The fifth row is the sum of alpha and beta and the sixth row the calculated pseudo *R*^2^ for all-interaction.

**Table 1:** Parameter values from the simulation for random interaction across cohorts.

*Cohort*	mean	1	2	3	4	5	6	7	8	9
*α*	0.7	0.5	0.5	0.8	0.8	0.7	0.7	0.6	0.9	0.7
*β*	0.8	0.9	0.9	0.8	0.8	0.8	0.9	0.8	0.5	0.9
Initial opinion (favor smoking)	0.02	0.02	0.02	0.02	0.02	0.02	0.015	0.02	0.019	0.01
Proportion	0.1	0.1	0.1	0.1	0.1	0.1	0.1	0.1	0.1	0.1
*α* + *β*	1.5	1.4	1.4	1.6	1.6	1.5	1.6	1.4	1.4	1.6
pseudo *R*^2^	0.99	0.97	0.98	0.99	0.99	0.98	0.98	0.98	0.96	0.99

**Fig 1:**
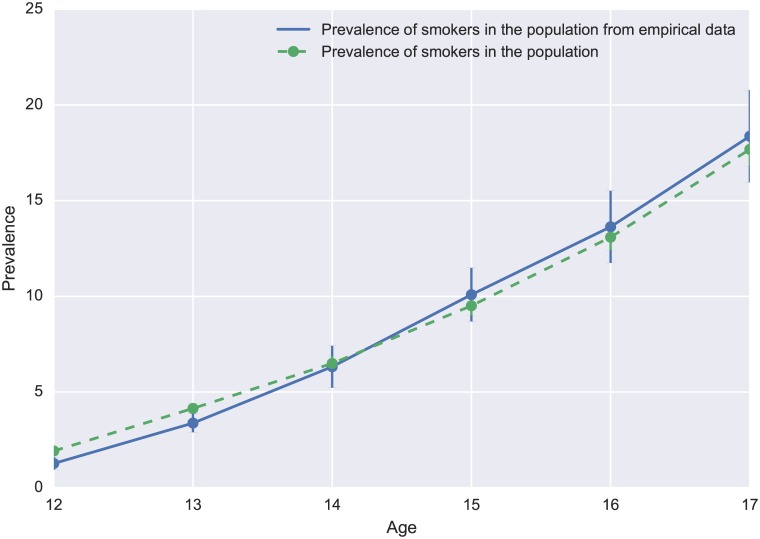
Simulated smoking prevalence using random interaction.

Fig 1 shows the simulated smoking prevalence using interaction rules against the empirical smoking prevalence using NSDUH data. From Fig 1 and calculated pseudo *R*^2^ for all cohorts in the table, we can see that our model adjusts well for NSDUH data. Pseudo *R*^2^ values are at least 96% and are consistent across all cohorts. Furthermore, the sum of and is greater than 1 in all cohorts.

**Fig 2:**
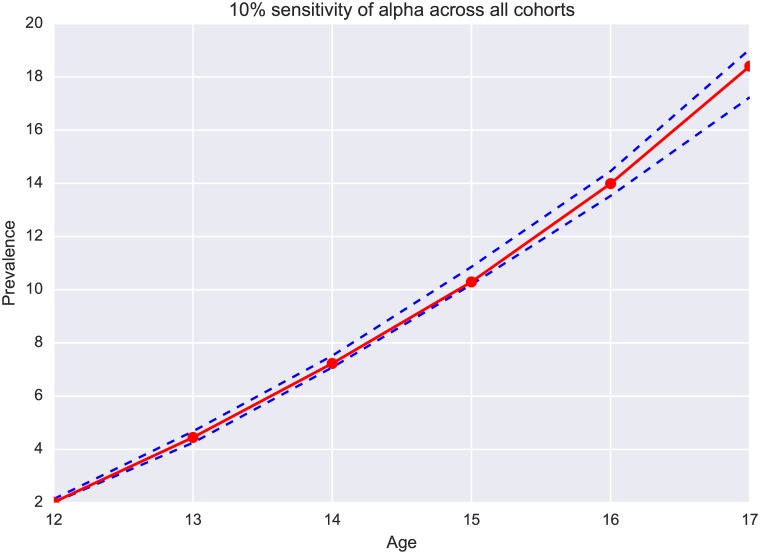
10% sensitivity of *α* across all cohorts.

**Fig 3:**
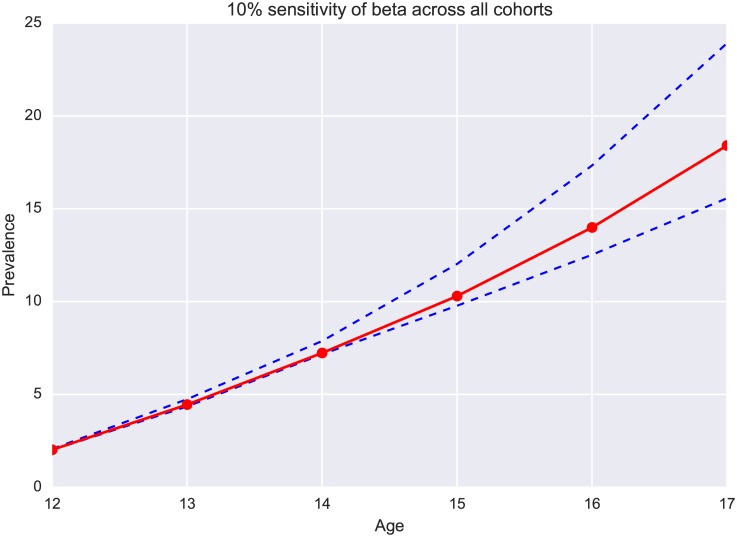
10% sensitivity of *β* across all cohorts.

Sensitivity analysis of likelihoods, *α* and *β*, are shown graphically in Figs 2 and 3. Here Fig 2 is the 10% sensitivity sweep of *α* for the average across 9 cohorts under random interaction and Fig 3 is the 10% sensitivity sweep of *β* for the average. These two graphs show that smoking prevalence is sensitive to changes in *α* and *β*. Given that our models fit the data well with optimal parameter values, we are confident that our estimates of these parameters provide unique information about the smoking initiation process.

### Parameter analysis

Based on the formula derived for *p*_*i*,*t*+1_, we can further derive some qualitative analysis of parameters *α* and *β* with respect to *p*_*i*,*t*+1_ to help interpret our simulation results.

We know there are three possible changes to from time *t* to time *t* + 1: 1) posterior opinion favoring smoking exceeds the original opinion (*p*_*i*,*t*+1_ > *p*_*i*,*t*_); 2) posterior opinion equals the original opinion (*p*_*i*,*t*+1_ = *p*_*i*,*t*_); 3) posterior opinion is below the original opinion (*p*_*i*,*t*+1_ < *p*_*i*,*t*_). Next we discuss the combination of and that leads to these three cases respectively.

If *j* is a smoker, from the earlier equation, we have

$$\begin{array}{c} p_{i,t+1}=\frac{O(f_{i,t}(S)\geq f_{i,t}(N)|\delta_{j,t}=+1)}{1+O(f_{i,t}(S)\geq f_{i,t}(N)|\delta_{j,t}=+1)}=\frac{\alpha p}{(1-\beta )(1-p)+\alpha p} \end{array}$$ (7)

1. *p*_*i*,*t*+1_ > *p*_*i*,*t*_
  ⇒ *α* > (1 − *β*) (1 − *p*) + *αp*
  ⇒ (*α* + *β*) (1 − *p*) > 1 − *p*
  ⇒ *α* + *β* > 1
2. *p*_*i*,*t*+1_ = *p*_*i*,*t*_
  ⇒ *α* = (1 − *β*) (1 − *p*) + *αp*
  ⇒ (*α* + *β*) (1 − *p*) = 1 − *p*
  ⇒ *α* + *β* = 1
3. *p*_*i*,*t*+1_ < *p*_*i*,*t*_
  ⇒ *α* < (1 − *β*) (1 − *p*) + *αp*
  ⇒ (*α* + *β*) (1 − *p*) < 1 − *p*
  ⇒ *α* + *β* < 1

If *j* is a non-smoker, we know

$$\begin{array}{c} p_{i,t+1}=\frac{O(f_{i,t}(S)\geq f_{i,t}(N)|\delta_{j,t}=-1)}{1+O(f_{i,t}(S)\geq f_{i,t}(N)|\delta_{j,t}=-1)}=\frac{(1-\alpha )p}{\beta (1-p)+(1-\alpha )p} \end{array}$$ (8)

1. *p*_*i*,*t*+1_ > *p*_*i*,*t*_
  ⇒ 1 − *α* > *β*(1 − *p*) + (1 − *α*)*p*
  ⇒ (1 − *α*) (1 − *p*) > *β*(1 − *p*)
  ⇒ *α* + *β* < 1
2. *p*_*i*,*t*+1_ = *p*_*i*,*t*_
  ⇒ 1 − *α* = *β*(1 − *p*) + (1 − *α*)*p*
  ⇒ (1 − *α*) (1 − *p*) = *β*(1 − *p*)
  ⇒ *α* + *β* = 1
3. *p*_*i*,*t*+1_ < *p*_*i*,*t*_
  ⇒ 1 − *α* < *β*(1 − *p*) + (1 − *α*)*p*
  ⇒ (1 − *α*) (1 − *p*) < *β*(1 − *p*)
  ⇒ *α* + *β* > 1

Our simulation results show that *α* + *β* > 1 hold for all cases. As a result, according to the qualitative analysis above, non-smokers increase their probability of smoking if they interact with a smoker and smokers decrease their probability of smoking if they interact with a non-smoker.

## Discussion

The sum of *α* and *β* has a threshold value of 1, where individuals exhibit different behaviors depending on the sum. If *α* + *β* < 1, probability of smoking decreases if a non-smoker interacts with a smoker and increases if a smoker interacts with a non-smoker. This is known as the contrarian behavior. Contrarians, according to Galam, are those who deliberately decide to oppose the prevailing choice of others [24].

On the other hand, if *α* + *β* > 1, the probability of smoking, *p*_*t*_, increases if a non-smoker interacts with a smoker and decreases if a smoker interacts with a non-smoker. The simulation results reveal that agents in our model are imitators, implying the existence of peer influence. Because we set *α*, *β* to be constant across individuals, the parameter values obtained describe the average behaviors of adolescents in the population.

Peer influence or imitation describes the phenomenon where peer interactions lead to convergence of behaviors. A few studies have found empirical evidence that support convergence of behaviors. The most well known is Christakis and Fowler’s Framingham Heart Study on spread of obesity [25]. The person-to-person spread of obesity they discovered indicates the existence of peer influence. Similar to obesity, peer influence has been found in smoking behaviors. Both Huang et al. and Lakon et al. investigated how peer influence affects smoking prevalence among adolescents [26, 27].

However, convergence of behaviors can result from two different mechanisms: peer selection vs peer influence. Peer selection, or homophily, refers to the process that people tend to become friends with those who are similar to them. And peer influence describes how friends develop similar behaviors. Agents who show convergence of behaviors could adopt both peer selection and peer influence mechanisms or either one. Unfortunately, our study only tests the hypothesis of imitation, aka peer influence. But our model adjusts well for NSDUH data, with pseudo *R*^2^ values to be at least 96% for nine different cohorts.

Teenagers exhibiting imitator behaviors has some theoretical implications for smoking initiation mechanism as well as policies. We know from previous work in opinion dynamics that contrarians can decreases the existence of extreme opinions while imitators tend to lead to extreme opinions [28]. If this imitator behavior would continue into adulthood, we will experience a continuously increasing prevalence of smoking, which we have not observed. We believe this imitator behavior does not continue into adulthood. Thus smoking policies targeting teenagers should be different from the ones for adults.

Besides implications for behavioral mechanism, our findings also offer some insights into smoking policies. Currently, most smoking policies target people as individuals and interventions usually focus on individual characteristics while ignoring the social network involved [29, 30]. This study demonstrates the importance of social network where individuals change their opinions and maybe smoking behaviors via social interaction.

In addition, imitation means that teenagers are likely to copy what their friends do. But smoking addiction as well as self-control issue can further complicate the measure of smoking prevalence among teenagers. If individuals form addiction to smoking, their behaviors are not necessarily consistent with their opinions. For example, person i with smoking addiction finds it very difficult to quit even with a strong opinion that favors non-smoking. But we know that teenagers are less likely to develop strong smoking addiction in their early ages due to barriers to smoking. Self-control can cast problem on the consistency between opinion and smoking in a similar manner. Our model allows for a probabilistic distribution of opinions to attenuate some of these effects.

Our study has some limitations. First, the CODA model implemented is relatively simple and does not take into account any external factors such as socioeconomic status (SES) or neighborhood effect. Second, we used a static social network structure (the grid) that does not allow any individual movement. In reality, teenagers make new friends and unfriend old ones from time to time. However, there is limited study on how exactly these processes take place. Lastly, we assumed in the CODA model that all agents share the same *α* and *β*. This assumption implies that the population is homogenous with the same likelihoods. For this study, we impose the homogeneity assumption because we are interested in the average behavior of the population. For future research, we plan to relax the assumption of homogeneity in model and impose more complicated social network structure that would allow for distributions of *α* and *β*.
